# Risk factors for COVID-19 among healthcare workers. A protocol for a systematic review and meta-analysis

**DOI:** 10.1371/journal.pone.0250958

**Published:** 2021-05-04

**Authors:** Tafadzwa Dzinamarira, Malizgani Mhango, Mathias Dzobo, Bernard Ngara, Itai Chitungo, Pelagia Makanda, James Atwine, Sphamandla Josias Nkambule, Godfrey Musuka

**Affiliations:** 1 Department of Public Health Medicine, School of Nursing and Public Health, University of KwaZulu-Natal, Durban, South Africa; 2 School of Public Health, University of Western Cape, Cape Town, South Africa; 3 Faculty of Medicine, College of Medicine and Health Sciences, University of Zimbabwe, Harare, Zimbabwe; 4 Department of Medicine, Jinzhou Medical University, Jinzhou, China; 5 ICAP at Columbia University, Harare, Zimbabwe; University of Oxford, UNITED KINGDOM

## Abstract

**Background:**

Evidence on the spectrum of risk factors for infection with severe acute respiratory syndrome coronavirus 2 (SARS-CoV-2) among front-line healthcare workers (HCWs) has not been well-described. While several studies evaluating the risk factors associated with SARS-CoV-2 infection among patient-facing and non-patient-facing front-line HCWs have been reported since the outbreak of the coronavirus disease in 2019 (COVID-19), and several more are still underway. There is, therefore, an immediate need for an ongoing, rigorous systematic review that continuously assesses the risk factors of SARS-CoV-2 infection among front-line HCWs.

**Objective:**

Here, we outline a protocol to serve as a guideline for conducting a living systematic review and meta-analysis to examine the burden of COVID-19 on front-line HCWs and identify risk factors for SARS-CoV-2 infection in patient-facing and non-patient-facing front-line HCWs.

**Methods:**

The protocol was developed and reported following the Preferred Reporting Items for Systematic Review and Meta-Analysis Protocols (PRISMA-P). The conduct of the proposed living systematic review and meta-analysis will primarily follow the principles recommended in the Centre for Reviews and Dissemination (CRD) guidance for undertaking systematic reviews in healthcare, and the Meta-analysis Of Observational Studies in Epidemiology (MOOSE) guidelines. The systematic literature searches will be performed using the EBSCOhost platform by searching the following databases within the platform: Academic search complete, health source: nursing/academic edition, CINAHL with full text, Embase, PubMed, MEDLINE, Science Direct databases, Google Scholar, and; also a search in the China National Knowledge Infrastructure and the World Health Organization library databases for relevant studies will be performed. The searches will include peer-reviewed articles, published in English and Mandarin language irrespective of publication year, evaluating the risk for testing positive for C0VID-19, the risk of developing symptoms associated with SARS-CoV-2 infection, or both, among front-line HCWs. The initial review period will consider articles published since the onset of COVID-19 disease to the present and then updated monthly. Review Manager (RevMan 5.3) will be used to pool the odds ratios or mean differences for individual risk factors where possible. Results will be presented as relative risks and 95% confidence intervals for dichotomous outcomes and mean differences, or standardised mean differences along with 95% confidence intervals, for continuous outcomes. The Newcastle–Ottawa Scale will be used to rate study quality, and the certainty of the evidence will be assessed by using the Grading of Recommendations, Assessment, Development and Evaluations (GRADE). The results of the living systematic review and meta-analysis will be reported per the PRISMA guidelines.

**Discussion:**

Though addressing the needs of front-line HCWs during the COVID-19 pandemic is a high priority, data to inform such initiatives are inadequate, particularly data on the risk factor disparities between patient-facing and non-patient-facing front-line HCWs. The proposed living systematic review and meta-analysis anticipate finding relevant studies reporting risk factors driving the SARS-CoV-2 infection rates among patient-facing and non-patient-facing front-line HCWs, thus providing subsidies for public health interventions and occupational health policies. The study results will be disseminated electronically, in print and through conference presentation, and key stakeholder meetings in the form of policy briefs.

**Trail registration:**

**PROSPERO registration number:** CRD42020193508 available for public comments via the link below https://www.crd.york.ac.uk/prospero/display_record.php?ID=CRD42020193508).

## Introduction

Pneumonia of unfamiliar aetiology was reported in December 2019 in Wuhan city in China and within a few months of its inception was declared a public health emergency of international concern by the World Health Organisation (WHO) [1]. The causative organism of the pneumonia was identified to be a novel member of the coronavirus family on the 7th of January 2020 [2]. On the 11th of February 2020 the WHO named the pneumonia coronavirus disease 2019 (COVID-19) whilst the International Committee on Taxonomy of Viruses named the novel virus severe respiratory Corona virus 2 (SARS-CoV 2) [1,2]. Human to human transmission of SARS-CoV-2 occurs through direct contact or air droplets by cough or sneeze from infected persons and the droplets can contaminate surfaces remaining infectious for several days in the environment providing a reservoir for the infection [1]. As of the 21^st^ February 2021, there have been more than 110 million confirmed infections and over 2,4 million confirmed deaths globally.

Respiratory pandemics are particularly virulent in their spread through droplets and interpersonal contact. Healthcare workers (HCWs), as front liners battling against the pandemic, are the most at risk of acquiring the infection as they are directly or indirectly exposed to infectious material. The COVID-19 pandemic has placed immense pressure on health systems and HCWs. Healthcare providers have to deal with a challenge of how best to distribute scarce resources equitably between HWCs and patients who are equally in dire need [3]. HCWs face an ever-present risk of acquiring or spreading the virus to their patients. A recently published rapid review identified lack of and/or inadequate personal protective equipment (PPE), exposure to infected patients, work overload, poor infection control and pre-existing medical conditions as important risk factors for nosocomial COVID-19 infection among HCWs [4]. However, a single rapid review can rarely validly assess the risk factors for SARS-CoV-2 infection among front-line HCWs, as new cases are reported daily.

At the time of initial writing of the protocol, in Italy, one of the COVID-19 worst-affected countries, as of the 25th of May 2020, 11.9% of all confirmed cases were HCWs [5]. On the 6th of May 2020, the International Council of Nurses reported that over 90 000 HCWs had been infected with COVID-19 [6]. A report published by the United States Centers for Disease Control and Prevention revealed that of the 9,282 infected HCWs in the Atlanta, Georgia between March and April 2020, 27 had died of the disease [7]. In Africa, the number of infected front-line workers have increased from 2,217 on the 26th of May to 4830 as of the 7th of June 2020 in 36 countries [8]. By August 15^th^, data collected from 37 countries found that nearly 300,000 healthcare workers had been infected with COVID-19 with over 2500 deaths [9]. However, the most relevant evidence to date is not conclusive as to the prevalence of risk factors for SARS-CoV-2 infection between patient-facing and non-patient-facing front-line HCWs according to specific clinical settings, thereby restricting the possibility of developing effective preventive measures to reduce occupational transmission of the virus, and consequently transmitting it to their household, workplace contacts across clinical settings, or both. Estimating the prevalence of risk factors in this population is important to guide public health measures to protect healthcare workers and their families, maintain a functioning healthcare system, and control rates of secondary transmission within the community. There is an urgent need to continuously surveil the literature and update the aggregated evidence base so that effective interventions, if such exist, are implemented clinically.

Therefore, a living systematic review allows us to incorporate relevant new emerging evidence, thereby decreasing the timespan from evidence to clinical practice, which is crucial in this international health crisis. The here proposed living systematic review with the meta-analyses, aims to continuously form the basis for evidence-based guideline recommendations for effective preventive measures to reduce occupational transmission of the virus; by identifying the spectrum of risk factors for SARS-CoV-2 infection, among front-line HCWs, taking bias risk (systematic errors), the play of chance (random errors), and certainty of the findings into consideration.

## Methodology

### Study design

The protocol is developed and reported in accordance with the reporting guideline provided in the Preferred Reporting Items for Systematic Reviews and Meta-Analysis Protocols (PRISMA-P) statement [10]. The protocol is registered with the International Registration of Systematic Reviews (PROSPERO 2020, CRD42020193508) and the published methodology is made available for public comments via the link below: https://www.crd.york.ac.uk/prospero/display_record.php?ID=CRD42020193508.

**Fig 1:**
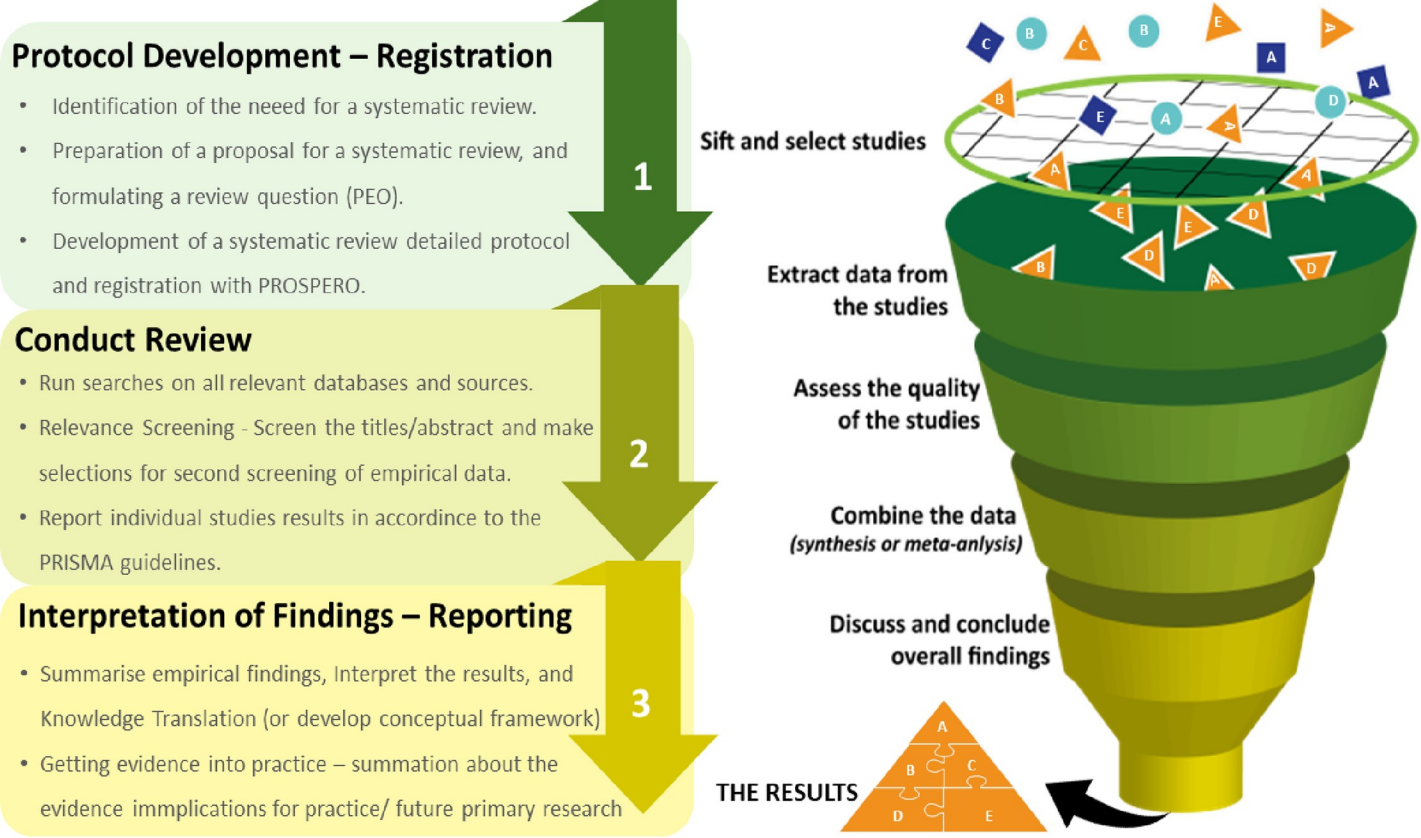
Schematic diagram of the seven steps of the methodological framework according to PRISMA. Source: Mulrow CD; 1994.

**Fig 2:**
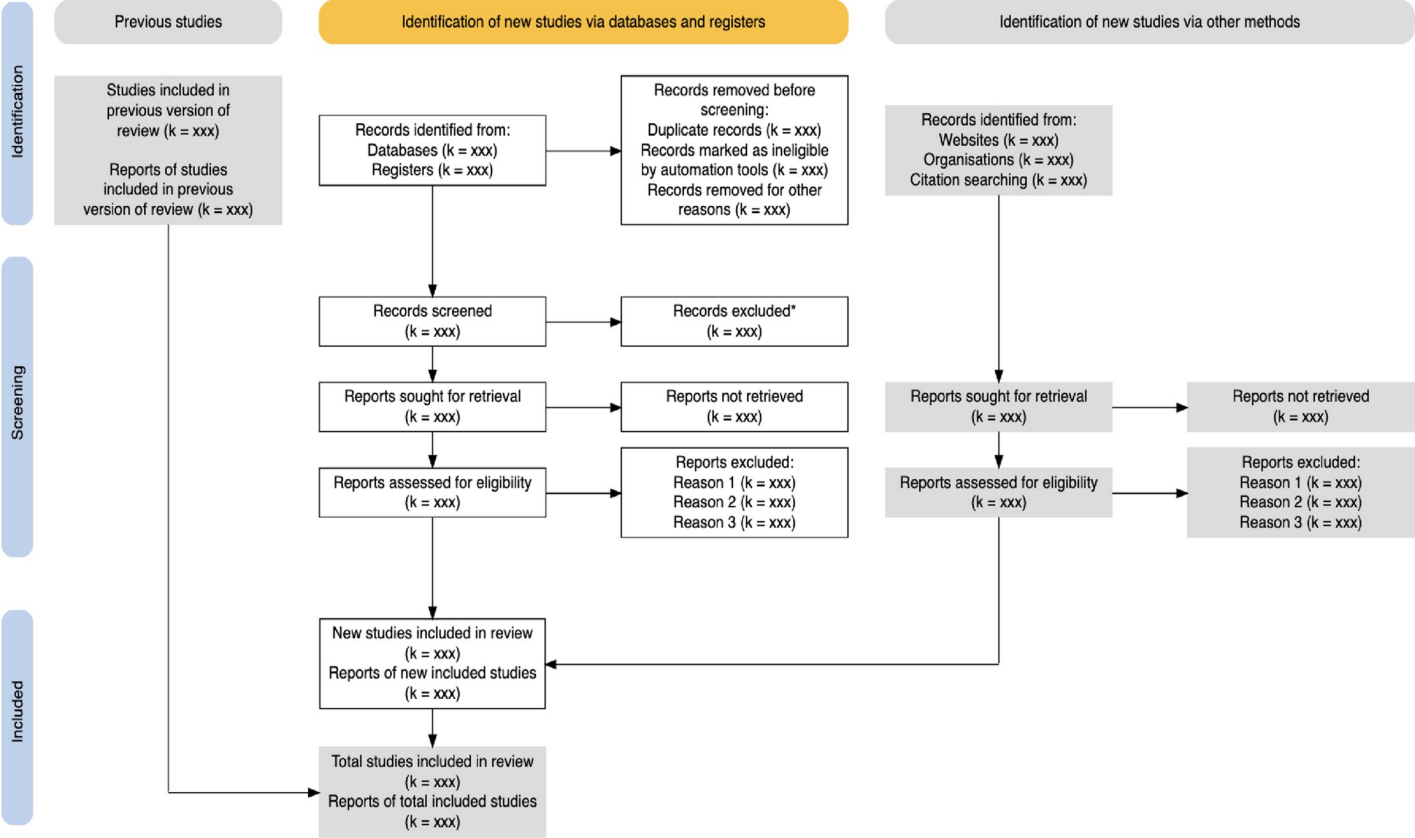
Preferred Reporting Items for Systematic Reviews and Meta-Analysis (PRISMA) 2020flow diagram of literature search and study selection process. Source: Adopted from Liberati A et al. 2009 [13].

The proposed living systematic review and meta-analysis will be carried out primarily, following the principles recommended in the Centre for Reviews and Dissemination (CRD) guidance for undertaking systematic reviews in healthcare [11], and additionally, following the Meta-analysis Of Observational Studies in Epidemiology (MOOSE) guidelines for design and implementation [12]. The results’ reporting would be consistent with the Preferred Reporting Items for Systematic Reviews and Meta-Analyses (PRISMA) guidelines [13] to ensure that the seven required steps are followed, as illustrated in **Fig 1** [14]. The PRISMA 2020 Flow Diagram [13,15] (**Fig 2)** will guide the flow of citations reviewed and illustrate the title search outcome from different databases. Lastly, the Newcastle–Ottawa Scale will be used to assess study methodological quality (or risk of bias) of all included studies [16], and the certainty of the evidence will be assessed by using the Grading of Recommendations, Assessment, Development and Evaluations (GRADE) guidelines [17].

The study seeks to identify and highlight relevant new literature or evidence as it becomes available on the distribution, trends and differences of risk factors for SARS-CoV-2 infection between patient-facing and non-patient-facing front-line HCWs, with estimates on the prevalence of COVID-19 among HCWs.

#### Step 1: Identifying the research question

To determine the research question’s eligibility for a systematic review project, we applied the PEO (Population, Exposure, and Outcomes) nomenclature for systematic reviews; recommended by the Joanna Briggs Institute 2015 [18,19]. The framework is illustrated in **Table 1**.

**Table 1:** PEO framework for determining the eligibility of the research questions.

Criteria	Determinant
Population (types of participants)	***Research Question 1 & 2***: Front-line HCWs* patient-facing and non-patient-facing at risk for or with SARS-CoV-2 infections, COVID-19 and any age groups, both genders, and regardless of their ethnic background country.
Exposure/ Risk Factor of interest (independent variable)	Research Question 1**: SARS-CoV-2 infections, positive for COVID-19, presence of symptoms associated with SARS-CoV-2 infection Research Question 2***: Demographic characteristics—age, sex, ethnic background, living circumstances (e.g. crowded housing, lack of separate rooms), self-quarantine methods Exposure history—in the workplace, home, or community, professional role/position, patient-facing and non-patient-facing front-line HCWs Administrative factors—policies; point of care assessment; patient flow/triage; usage of PEE training, adherence, availability of personal protective equipment; hours worked, shifts; contact hours; Health care setting and environment—unit worked (high-risk department e.g. ICU; lower risk, e.g. triage; etc.); institutional characteristics; use of negative pressure rooms; availability of hand hygiene stations HCW’s health—e.g., premorbid conditions/comorbidities; Infection prevention and control factors—policies, use (including reuse), training, adherence, availability, and type of personal protective equipment or handwashing; Others (as reported per study)
Outcomes (dependent variable)	***Research Question 1***: SARS-CoV-2 infection—Incidence, morbidity and mortality, social and economic effects of infection; and effects on family in exposed HCWs and infected HCWs ***Research Question 2***: Risk estimates (relative risk, odds ratio, or hazard ratio) for incidence or prevalence for risk factors; or incidence or prevalence reported by risk factor, Risk estimates and incidence of infections in household contacts of infected HCWs

Note

* HCWs—Definition of Health-Care Workers in this review is in accordance with the definition by the WHO [40].

The proposed living systematic review will address the following research question.

** Research Question 1—What is the burden of COVID-19 on front-line HCWs, and how do burden vary according to patient-facing and non-patient-facing front-line HCWs?.

*** Research Question 2—What are the risk factors for front-line HCWs infections with SARS-CoV-2, and how does the spectrum of risk factors vary according to patient-facing and non-patient-facing front-line HCWs?.

The proposed living systematic review will address the following research question;

1. What is the burden of COVID-19 on front-line HCWs, and how do burden vary according to patient-facing and non-patient-facing front-line HCWs?
2. What are the risk factors for front-line HCWs infections with SARS-CoV-2, and how does the spectrum of risk factors vary according to patient-facing and non-patient-facing front-line HCWs?

#### Step 2: Eligibility criteria for considering studies for inclusion in this review

The eligibility criteria are developed according to the relevant elements of the PEOd-T (Time) framework guidance for undertaking a systematic review [18,19] to ensure that the proposed living systematic review research question’s boundaries are clearly defined. Eligible studies will be included after two reviewers had independently, reproducibly, and systematically evaluated them; studies should present evidence on either of the factors, as illustrated in **Table 2**. Any disagreements between the two reviewers will be resolved by engaging a third reviewer.

**Table 2:** Eligibility criteria according to the PEOd-T nomenclature criteria.

Criteria	Inclusions	Exclusions
**Population**	Studies conducted in any part of the world involving front-line HCWs as participants; at risk for or with SARS-CoV-2 infections, COVID-19 irrespective of study setting will be eligible for inclusion Reported study participants will be included irrespective of gender, age, ethnicity, culture, race, comorbidities, patient-facing, or non-patient-facing or both	Research studies involving Non-HCW as participants
**Exposure/ Risk Factor of interest**	***Research Question 1***: Studies reporting a diagnosis of COVID-19 and the associated SARS-CoV-2 virus infection as an independent variable will be eligible; Studies reporting SARS-CoV-2 infections, positive for COVID-19, presence of symptoms associated with SARS-CoV-2 infection; 1) Confirmed by laboratory tests (such as reverse transcription-polymerase chain reaction (RT-PCR)); and 2) Assess positivity in relation to self-reported symptoms associated with SARS-CoV-2 infection will be eligible. Also, studies analysing the clinical characteristics and outcomes of laboratory-confirmed COVID-19 and the associated SARS-CoV-2 virus infection among HCWs. ***Research Question 2***: Studies examining the (1) demographic (2) occupational, and (3) Others (as reported per study) risk factors: 1) For testing positive for COVID-19, 2) the risk of developing symptoms associated with SARS-CoV-2 infection, or both, among front-line HCWs	Research studies reporting on other exposures/ risk factors.
**Outcomes**	***Research Question 1***: Studies reporting COVID-19 and the associated SARS-CoV-2 virus infection: 1) Incidence, morbidity and mortality, social and economic effects of infection as a dependent variable; and 2) Effects on a family in exposed HCWs, infected HCWs ***Research Question 2***: Studies reporting on one or more risk factors in HCWs will be eligible for inclusion. 1) Risk estimates (relative risk, odds ratio, or hazard ratio) for incidence or prevalence for risk factors; or incidence or prevalence reported by risk factor, as a dependent variable; Note: studies that report hazard ratio and those that report risk ratio will be treated separately. 2) Risk estimates and incidence of infections in household contacts of infected HCWs	Research studies reporting on other outcomes. None validated outcome measures
**Study Design**	Only original and quantitative study design that are peer-reviewed published in English and Mandarin language, irrespective of publication year will be eligible for inclusion Observational (cross-sectional, case-control, prospective and retrospective cohorts) and interventional (randomised controlled trials) epidemiological designs with front-line HCWs at risk for or with SARS-CoV-2 infections, COVID-19	Qualitative study designs, Systematic reviews (However, reference lists of relevant reviews will be checked for primary studies) Case reports, Anecdotal reports, and Modelling studies
**Time**	***No restrictions***–However, the initial review period will consider articles published since the onset of COVID-19 disease to the present and then updated monthly	

Search strategies with MeSH descriptors and truncation.

The PEO (Population, Exposure, and Outcomes) framework informed the development of the search strategy since the research question is more epidemiological and also to ensure that the boundaries of the research question are clearly defined. All searches were conducted on the 25th of June 2020.

Search restrictions and limitations

• Language = No Restrictions.

• Study Participants = Humans’ studies only.

Studies with outcomes deemed to be sufficiently homogenous will be eligible for inclusion in the meta-analysis; at least a minimum of two studies must report comparable quantitative data for exposure/ risk factor of interest, as illustrated in **Table 2**.

#### Step 3: Information sources and search strategy for the identification of relevant studies

*a) The literature search strategy*. The literature search for eligible studies for inclusion in the proposed review will be through a comprehensive and reproducible search of reputable bibliographic databases, indexing services (and platforms), and other supplementary information sources, such as Google Scholar and hand-searching [20]. All the primary searches, both electronic and hand-searching, will be performed simultaneously by two reviewers with a professional librarian’s assistance using a pre-defined search strategy.

The comprehensive search strategy has been co-developed by the first author, subject specialist and reviewed by all authors to ensure the correct use of indexing terminology and Medical Subject Headings (MeSH) descriptors. On the 25th of June 2020, the search strategy was pilot tested on a subset of records from the PubMed database and China National Knowledge Infrastructure (CKNI); details of the search strategies descriptors and truncation used, and the number of returned records are presented in **Table 3**. The search strategy keywords, MeSH descriptors and truncation, might be revised or updated before literature searches commence; this is chiefly because surveillance for new studies is ongoing. This process aims to obtain the maximum number of articles from the electronic databases, ensure that all relevant articles or reports are captured before the articles’ screening begins.

**Table 3:** Search strategy pilot search result from PubMed database and CKNI.

Date of Search	The search engine used (Database)	Keyword search used	No. of articles retrieved
	PubMed	("coronavirus"[MeSH Terms] OR "coronavirus"[All Fields]) AND ("COVID-19"[All Fields] OR "COVID-2019"[All Fields] OR "severe acute respiratory syndrome coronavirus 2"[Supplementary Concept] OR "severe acute respiratory syndrome coronavirus 2"[All Fields] OR "2019-nCoV"[All Fields] OR "SARS-CoV-2"[All Fields] OR "2019nCoV"[All Fields] OR (("Wuhan"[All Fields] AND ("coronavirus"[MeSH Terms] OR "coronavirus"[All Fields])) AND (2019/12[PDAT] OR 2020[PDAT]))) AND ("health"[MeSH Terms] OR "health"[All Fields]) AND ("occupational groups"[MeSH Terms] OR ("occupational"[All Fields] AND "groups"[All Fields]) OR "occupational groups"[All Fields] OR "worker"[All Fields]) AND ("health personnel"[MeSH Terms] OR ("health"[All Fields] AND "personnel"[All Fields]) OR "health personnel"[All Fields] OR ("health"[All Fields] AND "care"[All Fields] AND "worker"[All Fields]) OR "health care worker"[All Fields]) AND ("risk factors"[MeSH Terms] OR ("risk"[All Fields] AND "factors"[All Fields]) OR "risk factors"[All Fields] OR ("risk"[All Fields] AND "factor"[All Fields]) OR "risk factor"[All Fields])	637
	CKNI	Coronavirus 新型冠状病毒 OR Coronavirus infections 新型冠状病毒传染病 (or 大流行) OR Covid 19 2019年新型冠状病毒肺炎 infection 感染 Health Personnel 医务人员; 医疗人员 OR Health Workforce 医事人力资源 OR Humans 人类 Infectious diseases transmission 传染性疾病传播 Patient to professional 从患者到专业 (医务工作者) Occupational Exposure 职业性接触 (职业性暴露) OR Occupational health 职业卫生 (职业保健) Risk 风险 OR Risk Factors 风险因素	390

### Electronic searches

Advanced search strategies in the following electronic bibliographic databases (and platforms), comprising nine electronic databases, will be implemented to identify relevant studies: PubMed, Google Scholar, and EBSCOHost Web (Academic Search Complete, CINAHL Complete, MEDLINE with Full Text, CINAHL with Full Text, Health Source: Nursing/Academic Edition), China National Knowledge Infrastructure (CKNI) and WHO Global Database. All databases will be searched from their inception to the present, irrespective of publication language during the search strategy. However, only studies published in English and Mandarin will be included and reviewed. The reviewers will further browse through the link entitled ‘Related Articles’ option, which searches for similar citations using an intricate algorithm that scans titles, abstracts, and MeSH terms to detect more studies.

### Searching other resources–including hand searches

Systematic reviews are not eligible for inclusion; however, reference lists of relevant reviews, preprints, conference abstracts, and full-text articles will be screened for more relevant primary studies. The reference lists of relevant trial publications will be checked for any unidentified randomised clinical trials. The authors of the included trials will be contacted by email asking to provide all publicly releasable information about the clinical characteristics and outcomes of laboratory-confirmed COVID-19 and the associated SARS-CoV-2 virus infection among HCWs in randomised clinical trials of COVID-19. The COVID-19 Study Registry (https://covid-19.cochrane.org/) and COVID-evidence (https://covid-evidence.org/) will also be searched for more studies.

Furthermore, conference abstracts from COVID-19 conferences will be hand searched for relevant primary studies. Preprints identified from searching preprint servers (bioRxiv, medRxiv) will be screened for relevant primary studies.

b The search management. All retrieved articles from the electronic databases and hand searches, deemed to meet the inclusion criteria, will then be exported to EndNote X9 (version 19.1.0.12691)–a reference management software, which will be used to create a virtual library (Thomson Reuters, Stamford, CT, USA). Deduplication will follow immediately after transferring all the retrieved articles records to the Endnote X9. Moreover, the EndNote X9 virtual library will be created specifically for this study to manage records and data throughout the review and remove duplicates of the same records. We will use “Saving searches and Creating alerts platform on PubMed after running our searches strategies. A minimum 5 new studies alert would trigger reviewers to screen the new studies, and if new evidence is available (judged by the screeners’ steering committee), the results will be published. The updates would live on Zenodo, an open-access repository (https://zenodo.org/).

#### Step 4: Study selection and eligibility

The study selection is a multi-step process that involves two reviewers, namely MM and MD, for English databases and PM and JA for CKNI. The procedure for screening articles for eligibility will be carried out according to the Preferred Reporting Items for Systematic Reviews and Meta-Analysis (PRISMA) guidelines and illustrated in the PRISMA 2020 Flow Diagram **Fig 2** [12,13]. The study selection procedure will involve three screening stages:

Title screening. Firstly, the reviewers will search for eligible titles by analysing the titles of articles found through the search strategies’ use in the above mentioned electronic bibliographic databases (and platforms) guided by the study eligibility criteria (see **Table 2**). Articles with relevant titles will then be exported into the reference manager software–EndNote X9 (version 19.1.0.12691) virtual library. Duplicates will be removed before the start of the abstract screening stage.

Abstract screening. Following deduplication, two independent reviewers will begin in parallel the initial and abstract phase of screening. The titles and abstracts that do not meet the study eligibility criteria will be excluded. Differences between reviewers at this stage will be settled through reaching a consensus among reviewers.

Full-article screening. Following the abstract screening stage, full-text articles will then be retrieved where studies met the inclusion criteria or where there was ambiguity to be screened in greater depth by reference to the full-text assessment for eligibility. Thus, further establishing the retrieved studies’ eligibility. This stage will be conducted like the abstract screening stage by two independent reviewers (MM and MD, for English databases and PM and JA for CKNI). Studies that do not meet the inclusion criteria will be excluded. A secondary search of the reference list of all the included studies will follow immediately after the full-text screening; the two reviewers will carry it out for any articles that could not have been detected during the database search.

At this stage, the disagreements between the two reviewers will be assessed using Cohen’s Kappa coefficient (κ) statistic on Stata 13.0SE (StataCorp College Station, TX, USA), a robust statistic used for inter-rater reliability testing [21]. Detected differences between reviewers will be resolved by a third reviewer (TD), called to adjudicate discrepancies between the two reviewers.

All the included studies published in Mandarin retrieved from CKNI database will be translated into English by a review team member who is a native speaker for data extraction.

#### Step 5: Data extraction

After the two independent reviewers’ full-text screening stage, the data extraction will be performed on the included articles by two independent reviewers (MM and MD). The reviewers will extract data from the included studies separately and in-duplicate to detect inter-rater errors and reduce data errors and bias. The data extraction procedure will be facilitated by a standardised data abstraction form (Additional File 1), two reviewers working independently and in duplicate will extract essential data into the standardised tables, and a third reviewer will verify the data related to study characteristics: study author, year, setting (country, health care setting, dates), population characteristics (sample size, age, gender, HCW role/position, number of cases), and results. The outcome measures to be analysed for main research question one and two are stipulated in **Table 2**.

In the meta-analysis, odds ratios (ORs) will be calculated if necessary, and if the data is available, only measures most adjusted for one or more sets of potential confounders such as socio-demographic and lifestyle factors will be extracted to reduce confounding and measurement errors, and to ensure consistency across studies and reduce bias.

Authors of included studies will be contacted to obtain missing information or clarify information on methodology (e.g. study setting, case definition) and outcomes. In cases of non-response or inadequate clarity from contacted authors (i.e. retrieval of missing data is not possible), that study/ outcome will be excluded from the review.

#### Step 6: Assessment of study quality and risk of bias

The Newcastle–Ottawa Scale (NOS) [16] will be used to rate study quality. The NOS allows assessment of the quality of design of non-randomised case-control and cohort studies. The scores will be assigned for selection criteria, comparability and outcome (cohort) or exposure (case-control). Two reviewers will assess methodological quality (MM and MD). Discrepancies will be resolved by a third reviewer (TD).

The overall quality of evidence (or certainty in the findings) for each outcome collected will be assessed using the five GRADE considerations (bias risk of the trials, consistency of effect, imprecision, indirectness, and publication bias). The completed GRADE checklist and reasons to up-or down-grade assess the quality of a body of evidence, based on study methodological quality, results from sensitivity analysis, and by downgrading and upgrading the baseline quality score according to the domains specified in the *grades of recommendation*, *assessment*, *development*, *and evaluation* (GRADE) guidelines [17]. We will produce a Summary of Findings table.

#### Step 7: Data synthesis and analysis of results

The proposed living systematic review and meta-analysis aim to integrate new data on the burden of COVID-19 and the variations in the risk factors for SARS-CoV-2 infection between patient-facing and non-patient-facing front-line HCWs, with estimates on the prevalence of COVID-19 and risk factors among front-line HCWs.

Firstly, after completing the data extraction step, a descriptive numerical account of the extracted data from the included studies will be generated and presented in a compiled summary of findings table. The descriptive summary of the findings table will include each of the prespecified outcomes (see **Table 2**), and details of the characteristics of the included studies, such as the total number of publications included, type of study design, year of publication, type of risk factors reported, characteristics of the study populations, and study setting.

Secondly, Canva version 2.93.0 [22]—an interactive web-based graphic design tool–will then be used to model a world map to visualise the variation patterns in the occurrence, distribution and trends in the burden of and risk factors for SARS-CoV-2 infections among HCWs in the world. Thirdly, tables and figures will be used to present the results in an organised way to satisfy the two main research questions addressed by the proposed living systematic review and meta-analysis.

- *Research question one—what is the burden of COVID-19 on front-line HCWs*, *and how do burden vary according to patient-facing and non-patient-facing front-line HCWs*?. Because of the type of data sought to satisfy research question one and, most probably due to anticipated methodological limitations, study design variability, heterogeneity in populations, comparisons, and analytic methods across the included studies, the findings will be synthesised narratively.
- *What are the risk factors for front-line HCWs infections with SARS-CoV-2*, *and how does the spectrum of risk factors vary according to patient-facing and non-patient-facing front-line HCWs*?. Quantitative data will be extracted from the included studies to determine the prevalence of and risk factors for SARS-CoV-2 infections among patient-facing and non-patient-facing front-line HCWs. A similar type of risk factors for SARS-CoV-2 infections will be pooled from each study for overall estimates. The categories of risk factors (i.e., demographic, occupational, and others) reported from all included studies will be synthesised descriptively to understand the key risk factors for SARS-CoV-2 infections among patient-facing and non-patient-facing front-line HCWs.
- *Statistical analysis*. The prevalence estimates of SARS-CoV-2 infections among patient-facing and non-patient-facing front-line HCWs will be calculated by meta-analysis. However, studies with a low risk of bias and outcomes deemed to be sufficiently homogenous; will be selected for inclusion in the meta-analysis to compute the pooled prevalence estimates.

The pooled prevalence of SARS-CoV-2 infections will be estimated from the reported prevalence of eligible studies using the statistical software Review Manager (RevMan) V.5.2.1 (The Nordic Cochrane Centre, Copenhagen, Denmark). Forest plots will then be generated, displaying prevalence with the corresponding 95 per cent confidence interval (CI) for each study [23], analysed using the random-effects model, utilising the inverse variance method of the DerSimonian-Laird approach to estimate the heterogeneity between-study population [24].

For research question two, meta‐analysis will be used to estimate the pooled odds ratio (OR) for categorical data, or mean difference (MD) for continuous data, between healthcare workers with COVID-19 and those without, for reported risk factors. We expect outcomes such as Age in years (continuous), Townsend deprivation score (continuous), and also prognostic risk factors for disease severity and death in terms of laboratory results. For example; increased lactate dehydrogenase (LDH), C-reactive protein (CRP), decreased blood platelet, lymphocytes count, and elevated D-dimer levels as many emerging descriptive studies have reported a correlation measured on admission with disease severity and increased risk of death in patients with COVID-19. A metaregression of the key risk factors’ ORs will then be conducted to identify each risk factor’s individual effects for SARS-CoV-2 infections among front-line HCWs. The listed covariates below were selected from peer-reviewed rapid and systematic reviews [4,25], epidemiological studies [26], and authoritative sources [27], including reviewing the evidence on the World Health Organization COVID-19 Situation Reports [1] for known or established risk factors of the outcome in the general population.

The list of covariates that will be used in the meta-regression:

1. Old age, male, smoking, high body mass index, high breathing rate; and
2. A combination of underlying diseases (any comorbidity such as hypertension, diabetes, cardiovascular disease, and chronic obstructive pulmonary disease, especially chronic kidney disease, cerebrovascular disease);
3. Geographic region, study period, public awareness.

To reduce confounding and measurement errors, and ensure consistency across studies and reduce bias, only adjusted risk estimates will be presented for the synthesis. The overall random-effects pooled estimate with its CI will be reported for both patient-facing and non-patient-facing front-line HCWs.

d *Assessment of heterogeneity*. In the meta-analysis, heterogeneity is unavoidable due to differences in study quality, sample size, method and outcome measurement across studies. Therefore, the forest plots will be examined in the first place to determine some evidence of heterogeneity visually [28].

Secondly, if any signs of heterogeneity are detected from examining the forest plots; the I^2^ statistics for statistical significance of heterogeneity and its CI (i.e., the percentage of variance not due to studies-wide sampling error) will be used to calculate the magnitude of the heterogeneity between studies included in all meta-analyses [28–30]. Consequently, the following I^2^ cut-offs for low, moderate and high heterogeneity will be used to report the degree of heterogeneity, as per the guidelines of the *Cochrane Handbook for Systematic Reviews of Interventions* [31]: 1—between 0% to 40% = might not be important; 2–30% to 60% = may represent moderate heterogeneity; 3–50% to 90% = may represent substantial heterogeneity; and 4–75% to 100% = considerable heterogeneity.

Furthermore, subgroup analyses will be conducted to examine evidence heterogeneity (see ‘Subgroup analyses and integration of heterogeneity’ below). In the end, we can determine that a meta-analysis should be avoided if heterogeneity is large [29].

e *Assessment of reporting biases*. Moreover, reporting bias (Meta-biases) will be assessed using the funnel plots to detect potential reporting biases and small-study effects [32]; if more than ten studies are included in the meta-analysis. The funnel plots will be visually inspected for asymmetry (a plot of effect estimates against sample sizes), complemented with the Egger regression test. The graph’s symmetrical shape is interpreted as the absence of publication bias, while an asymmetrical shape of the graph is interpreted as the possibility of publication bias [31,33].
f *Subgroup analysis and integration of heterogeneity*. Subgroup analyses will be performed to evaluate the influence of the different study characteristics: publication year, setting (country, health care setting, dates), population characteristics (age, gender, HCW role/position, number of cases). The most commonly used age bands for consultation with adults will be employed. For instance, the most commonly used bands (18–24, 25–34, 35–44, 45–54, 55–64 and 65 and over) will be used as age cut-offs in the subgroup analysis. However, we note the uncertainties in carrying out subgroup analyses as not all studies will stratify their results by age and gender, or may use different thresholds.

Stratified prevalence will be generated according to demographic characteristics, study setting, patient-facing and non-patient-facing front-line HCWs, country’s economic levels (low income, lower middle income and upper middle income), publication year and by sampling methods (random and convenience).

g *Sensitivity analysis*. Sensitivity analyses will be performed to assess the meta-analysis results’ stability, verify the robustness of the study conclusions, and further evaluate the potential influence of single studies on the pooled prevalence estimates by sequential omission of one study at a time. We will also use sensitivity analyses to investigate suspected funnel plot asymmetry due to publication bias if any.

### Differences between the protocol and the review

According to this published protocol, we shall perform a review and note any discrepancies from it by updating the PROSPERO record and detailing changes in a “Differences between the protocol and the review” section of the published systematic review results article.

### Ethics and dissemination

No ethical clearance is required for this study. The results of the proposed living systematic review and meta-analysis will be disseminated electronically, in print and through conference presentation as well as key stakeholder meetings.

## Discussion

A living systematic review is described as a systematic review that is constantly revised and integrates the relevant new evidence as it becomes accessible [34]. This evidence synthesis methodological approach may be especially relevant in the context of the COVID-19 pandemic, where scientific evidence is evolving rapidly, existing evidence is unclear, and new evidence is highly sought for that may alter policy or practice decisions [34,35]. This systematic review will provide useful information to researchers, policymakers, and healthcare providers on the risk factors that predispose HCWs to COVID-19 infection. This information is important in the formulation of occupational health policies for HCWs in the context of the COVID-19 pandemic and future outbreaks of similar nature. With the pandemic still rapidly increasing in some parts of the world, the protection of HCWs is key as they are at the forefront of fighting the pandemic.

There are four basic distinctions between traditional systematic reviews and living systematic reviews: publishing format, work processes, author team management, and statistical approaches [19]. In this living systematic review, we adopted the Cochrane standard workflow tool to maximize quality and efficiency in the review process [36,37]. The Cochrane standard workflow tool provides a framework for managing review production and setting expectations about responsibilities and timelines at the outset of the review.

The living systematic review process will be initiated as soon as the protocol is accepted for publication. The initial review period will consider articles published since the onset of COVID-19 disease to the present date and then updated monthly. Given that many studies related to the research question are expected to be published in the following months, coupled with the urgency to have the most up-to-date evidence as soon as possible–every month, the steering committee will discuss whether searching once a week is necessary [34,38–40]. The meta-analyses will continuously be updated, and if new evidence is available (judged by the steering committee), the results will be published.

There are chances of considerable under-reporting on the risk factors in our study particularly since the disease is spreading quickly with new hotspots emerging in different geographical areas of the world. This systematic review is limited to English and Mandarin publications only. This may not cover other important studies published in other languages from other affected parts of the world. The inclusion of studies published in Mandarin is a strength of this review as China is the source country of the COVID-19 pandemic hence the likelihood of a significant number of published articles on the research question.

## Supporting information

S1 Checklist PRISMA-P (Preferred Reporting Items for Systematic review and Meta-Analysis Protocols) 2015 checklist: Recommended items to address in a systematic review protocol. (DOC)

S1 File (XLSX)
